# A protein folding molecular imaging biosensor monitors the effects of drugs that restore mutant p53 structure and its downstream function in glioblastoma cells

**DOI:** 10.18632/oncotarget.25138

**Published:** 2018-04-20

**Authors:** Ramasamy Paulmurugan, Rayhaneh Afjei, Thillai V. Sekar, Husam A. Babikir, Tarik F. Massoud

**Affiliations:** ^1^ Cellular Pathway Imaging Laboratory (CPIL), Molecular Imaging Program at Stanford, Stanford University School of Medicine, Palo Alto, CA 94305, USA; ^2^ Laboratory for Experimental and Molecular Neuroimaging (LEMNI), Molecular Imaging Program at Stanford, Stanford University School of Medicine, Stanford, CA 94305, USA

**Keywords:** drug screening, luciferase, misfolding, Renilla, temozolomide

## Abstract

Misfolding mutations in the DNA-binding domain of p53 alter its conformation, affecting the efficiency with which it binds to chromatin to regulate target gene expression and cell cycle checkpoint functions in many cancers, including glioblastoma. Small molecule drugs that recover misfolded p53 structure and function may improve chemotherapy by activating p53-mediated senescence. We constructed and optimized a split *Renilla* luciferase (RLUC) complementation molecular biosensor (NRLUC-p53-CRLUC) to determine small molecule-meditated folding changes in p53 protein. After initial evaluation of the biosensor in three different cells lines, we engineered endogenously p53^P98L^ mutant (i.e. not affecting the DNA-binding domain) Ln229 glioblastoma cells, to express the biosensor containing one of four different p53 proteins: p53^wt^, p53^Y220C^, p53^G245S^ and p53^R282W^. We evaluated the consequent phenotypic changes in these four variant cells as well as the parental cells after exposure to PhiKan083 and SCH529074, drugs previously reported to activate mutant p53 folding. Specifically, we measured induced RLUC complementation and consequent therapeutic response. Upon stable transduction with the p53 biosensors, we demonstrated that these originally p53^P98L^ Ln229 cells had acquired p53 cellular phenotypes representative of each p53 protein expressed within the biosensor fusion protein. In these engineered variants we found a differential drug response when treated with doxorubicin and temozolomide, either independently or in combination with PhiKan083 or SCH529074. We thus developed a molecular imaging complementation biosensor that mimics endogenous p53 function for use in future applications to screen novel or repurposed drugs that counter the effects of misfolding mutations responsible for oncogenic structural changes in p53.

## INTRODUCTION

Proteins catalyze, facilitate and regulate all the biological processes that occur in living systems [1]. The three-dimensional structure of a protein defines its function [2, 3]. There are many protein misfolding-associated human diseases, e.g. Alzheimer's disease (APP and Tau), Parkinson's disease (ɑ-synuclein), Huntington's disease (Huntingtin), and cystic fibrosis (CFTR protein) [4], to name a few. Moreover, human cancers may arise as a phenotypic consequence of accumulated somatic mutations that result either from loss of a group of proteins, or, loss- or gain-of-function consequent to protein structure perturbations [5]. P53, the guardian of the genome, is considered an important tumor suppressor protein in cells; the functional loss of this protein in part or as a whole can create significant cellular changes leading to cancer. In particular, amino acid substitutions in the DNA-binding domain of p53 are a frequent occurrence in many types of cancer and, therefore, an attractive target for therapeutic interventions [6]. Drugs that stabilize p53 by preventing its ubiquitination or its interaction with human double minute 2 homolog (HDM2) protein is a possible approach to enhance chemosensitivity in cancers that are endogenously single allele deletion/mutant for p53 expression [7]. By contrast, drugs that rescue protein function by stabilizing mutant proteins are currently under investigation in several disease contexts and include pharmacoperones: small molecules that function as pharmacological chaperones [8]. Approaches that could enable real-time noninvasive monitoring of this rescue process by detecting changes in protein folding within intact cells would be especially useful for identifying and validating new drugs that improve cancer therapy.

We previously developed protein-fragment complementation assays (PCAs) using various reporter proteins [9–15]. We studied these PCAs by measuring both protein-protein interactions and ligand-induced structural changes of proteins in cells and living subjects [9–15]. Examples include evaluation of the structural changes induced by estradiol and its analogs to the estrogen receptor (ER) [9], ligand mediated homo- and heterodimerization of ERs, rapamycin mediated heterodimerization of FRB and FKBP12, and several other protein-protein interaction partners [10, 11]. We also used PCAs to screen drugs that inhibit Hsp90 interaction with p23 protein, and phosphorylation of protein kinase C [12–14]. Importantly, we recently demonstrated the use of a PCA in monitoring protein folding and mutation-induced collapse of the chromophore structure in enhanced green fluorescent protein within cells [15].

Herein, we exploit the close proximity of the amino and carboxyl termini of native properly folded proteins to drive a protein folding PCA [9, 15] that can indirectly predict the presence of a mutation in the DNA-binding domain of p53 protein, and in which single amino acid substitutions have been shown to induce structural changes and functional loss of this protein in cells. We screen Firefly luciferase (FLUC) and *Renilla* luciferase (RLUC) PCAs generated using different reporter protein split sites and identify optimal PCAs that efficiently detect structural changes in p53. We then evaluate these PCAs using drugs that recover and reactivate p53 function in cells. We further study the functional effects of these drugs in enhancing p53-mediated apoptotic pathways in cells by co-treating with the clinically relevant chemotherapeutic drugs, doxorubicin (Dox) and temozolomide (TMZ). Using this PCA, we demonstrate efficient activation of p53 structural alteration-mediated luciferase complementation in cells, and validate the potential role of small molecules as combination chemotherapeutic agents for treatment of glioblastoma cells engineered to express PCA biosensors containing different p53 mutants. This generalizable methodology is highly useful to image proteins in real-time in intact cells (and also potentially in living subjects) to screen for drugs that reactivate p53 function.

## RESULTS

### Identification of a complementation biosensor based on split-*Renilla* luciferase (at residue 229) as being optimal for measuring structural folding changes in p53 protein carrying structural mutations in its DNA-binding domain

To enable a split-luciferase complementation assay that can efficiently detect mutation-induced structural folding changes in p53 protein, we first constructed fusion proteins of p53 or its mutants (p53^wt^, p53^Y220C^, p53^G245S^ and p53^R282W^) with N- and C- terminal luciferase fragments of Firefly luciferase (N-398/C-394) or *Renilla* luciferase (N-229/C-229 and N-110/C-110) (Supplementary Figures 1 & 2). To determine the effects of protein fragment complementation on the function of p53, we transiently transfected all four sensors in HEK293T cells and assessed luciferase activity after 24 h. We found significant complementation assisted luciferase signal from all constructs, but the biosensor containing FLUC fragments showed no significant difference between constructs, while the RLUC biosensor with a split site at residue 110 led to a low level of signal with wt-p53 compared to mutants (Supplementary Figure 3). Of note, biosensors with the RLUC complementation system using a split-site at residue 229 showed significantly higher complementation signal for wt-p53 protein compared to biosensors with mutant p53 proteins (Supplementary Figure 3B). We also tested the consistency of RLUC biosensor in two other cell lines (Ln229, and U87MG [Supplementary Figure 3C]) with different genetic backgrounds by transient transfection. We also tested the response to PhiKan083 in cells transfected with the FLUC complementation system and found no significant improvement in the signal intensity after drug treatment (Supplementary Figure 3D). We thus identified an optimal split-RLUC PCA system that can detect and quantify refolding of p53 protein in cells.

### Use of Ln229 cells stably expressing equal levels of complementation biosensors tailored to different p53 proteins confirmed their specificity in detecting structural folding changes in p53 protein carrying structural mutations in its DNA-binding domain

**Figure 1:**
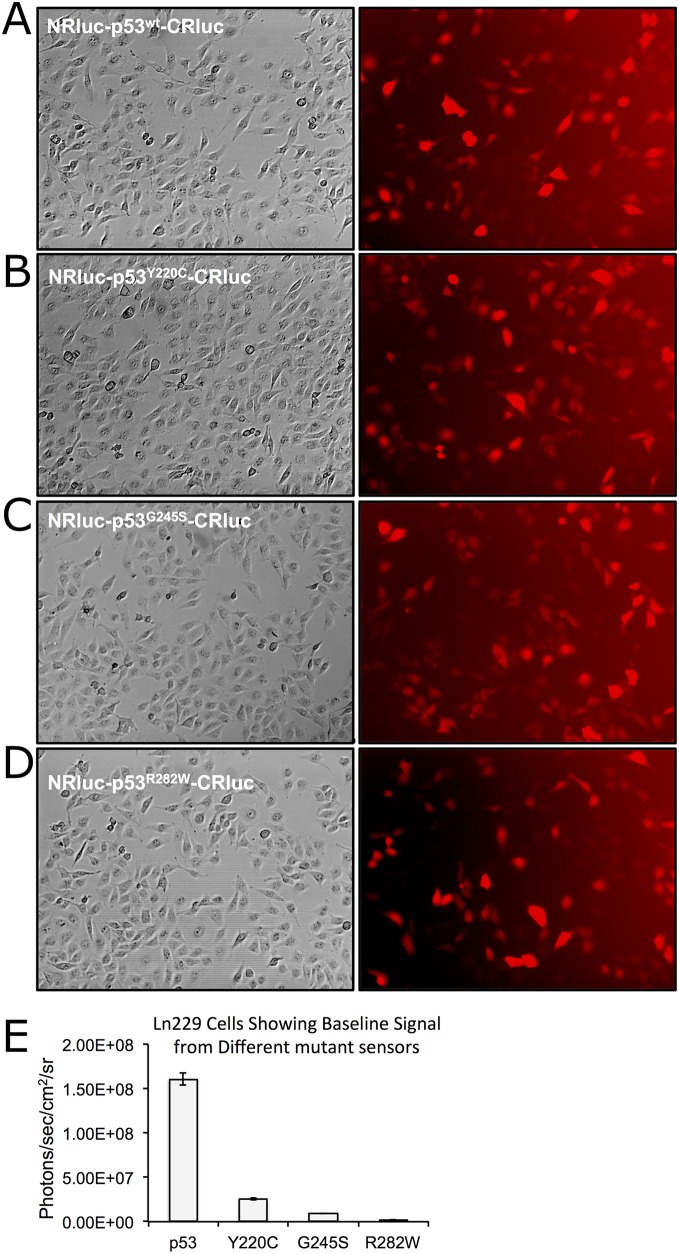
Clonal population of Ln229 cells engineered to stably express split-*Renilla* luciferase complementation sensors with different p53 proteins (p53^wt^, p53^Y220C^, p53^G245S^, p53^R282W^) at equal levels and tested for the basal level of protein folding-assisted luciferase complementation signal using bioluminescence imaging **(A-D)** Bright field and respective dTomato fluorescent images of Ln229 variants expressing different complementation biosensors. **(E)** Graph showing the bioluminescent complementation signal measured from different clones.

After establishing the optimal complementation system we developed lentiviral vectors (Supplementary Figure 2) expressing these biosensors to help generate stable Ln229 cells expressing different complementation biosensors. We sorted these stable cells to select clonal populations with equal extents of vector integration using the co-expressed dTomato fluorescent protein (Figure 1A-1D). We tested the engineered cells for evaluating luciferase complementation, and found that the cells expressing wt-53 biosensors showed significantly higher levels of luciferase complementation compared to cells expressing biosensors with mutant-p53 proteins (Figure 1E).

### Ln229 cells stably expressing equal levels of complementation biosensors tailored to different p53 proteins measured drug-induced structural changes in p53 proteins

**Figure 2:**
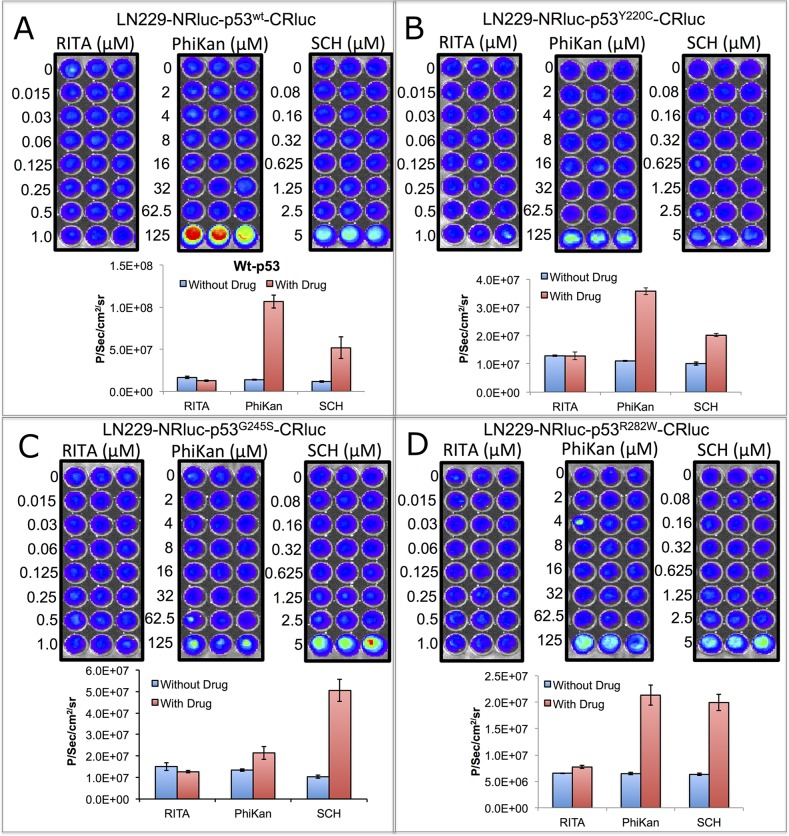
Imaging split *Renilla* luciferase complementation induced by drugs (PhiKan083 and SCH529074) that specifically bind to p53 protein and induce structural change in mutant-p53 to acquire wild-type or near wild-type conformation Ln229 cells stably engineered to express RLUC complementation sensor with p53^wt^ **(A)**, p53^Y220C^ **(B)**, p53^G245S^ **(C)** and p53^R282W^ **(D)** treated with different concentrations of RITA (non-binder), PhiKan083 and SCH529074, imaged for activated luciferase complementation signal. Images are shown in the top and respective quantitative graphs in the bottom.

**Figure 3:**
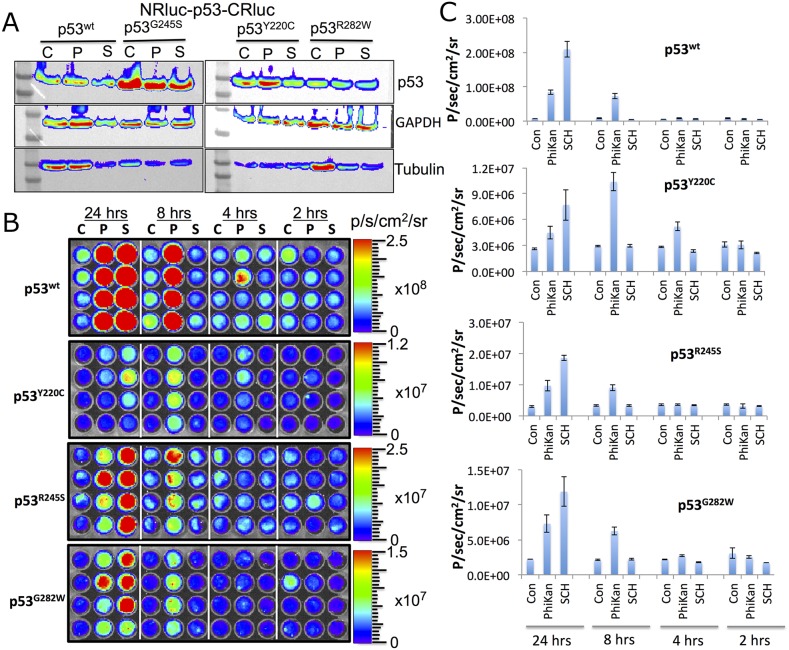
**(A)** Immunoblot analysis of Ln229 cells engineered to stably express split-*Renilla* luciferase complementation sensor with different p53 variants after treating with PhiKan083 (50 μM) and SCH529074 (1.25 μM) for 24 h. **(B)** RLUC complementation signal measured in Ln229 cells expressing different p53-variants (p53^wt^, p53^Y220C^, p53^G245S^, p53^R282W^) after treating with PhiKan083 and SCH529074 for various durations (C: Control, P: PhiKan083 and S: SCH529074). **(C)** Quantitative graphs of bioluminescence signals measured from images shown in (B).

Next, we tested structural changes in p53 protein induced by the drugs PhiKan083 and SCH529074 in Ln229 cells stably expressing the biosensors. We treated the cells for 16 h with respective drugs in different concentrations based on their relative binding affinity (RBA) to p53 protein (PhiKan083, RBA to p53^Y220C^ is 150 μM; SCH529074, RBA to p53^N268R^ is 2 μM; RBAs for other mutants are not available in the literature). We also used RITA, a small molecule drug previously reported to stabilize p53 protein by blocking its interaction with HDM2 mediated ubiquitination. We found selective activation of p53-mediated luciferase complementation by both PhiKan083 and SCH529074 when these drugs were at concentrations closer to their RBAs, while RITA showed no significant change in luciferase signal (Figure 2). PhiKan083 revealed significantly higher levels of activation at 125 μM in cells expressing p53^Y220C^ biosensor compared to p53^G245S^. Interestingly PhiKan083 induced significant levels of complementation signal in cells expressing p53^wt^ and p53^R282W^ biosensors (Figure 2). To check that this increase in observed complementation signal was not merely owing to an increase in quantity of available p53 protein through stabilization, we performed immunoblot analysis using p53 antibody. There was no significant increase in p53 levels in response to treatment (Figure 3A). We also tested to see the time points at which the drugs were able to induce maximum levels of luciferase complementation compared to controls. Thus, we treated Ln229 cells stably expressing the biosensors with 125 μM PhiKan083 and 2.5 μM SCH529074 for 2, 4, 8 and 24 h. The cells analyzed by optical imaging showed significant levels of activation as early as 4 h after drug treatment, with a maximum level at 24 h (Figure 3B & 3C).

### Ln229 cells engineered to stably express the p53 folding biosensors demonstrated differential phenotype-associated variation in therapeutic response to Dox and TMZ compared to parental cells

**Figure 4:**
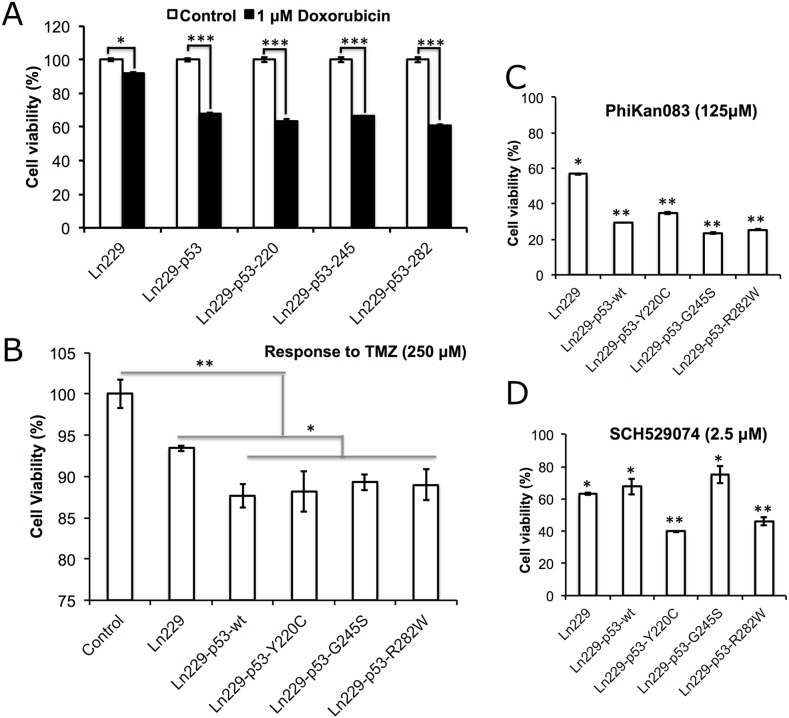
Therapeutic response of Ln229 cells stably expressing split-*Renilla* luciferase complementation sensor with different p53 variants (p53^wt^, p53^Y220C^, p53^G245S^, p53^R282W^) in response to the treatment of **(A)** Doxorubicin (1 μM), **(B)** Temozolomide (250 μM), **(C)** PhiKan083 (125 μM) and **(D)** SCH529074 (2.5 μM), for the change in cell viability and induced apoptosis.

We evaluated the therapeutic response of Ln229 cells stably expressing the p53 folding biosensors to Dox and TMZ, and also in response to treatment using PhiKan083 and SCH529074. Cells treated with 1 μM Dox, 250 μM TMZ, 125 μM PhiKan083 and 2.5 μM SCH529074 were tested for induced cell growth arrest 48 h after treatment using an MTT assay. We found a significant response from all four engineered clones to Dox compared to parental Ln229 cells (~40% reduction in cell viability in the engineered clones, compared to only <10% reduction in the parental cells) (Figure 4A). Similarly, we observed significant enhancement in treatment response from engineered p53 variants of Ln229 cells to TMZ compared to the parental cells (Figure 4B). Independently, we also tested their response to 125 μM PhiKan083 and observed ~70 ± 5% reduction in cell viability when using engineered variants of Ln229 cells compared to parental Ln229 cells (Figure 4C). In contrast, treatment using 2.5 μM SCH529074 resulted in selective augmentation of cell growth arrest in cells expressing p53^Y220C^ and p53^R282W^ (~60%), compared to parental cells and variants expressing p53^wt^ and p53^G245S^ (~40%) (Figure 4D).

### Treatment of engineered variants of Ln229 cells to Dox alone or in combination with PhiKan083 showed altered therapeutic response through p53 folding instead of improving protein stabilization

**Figure 5:**
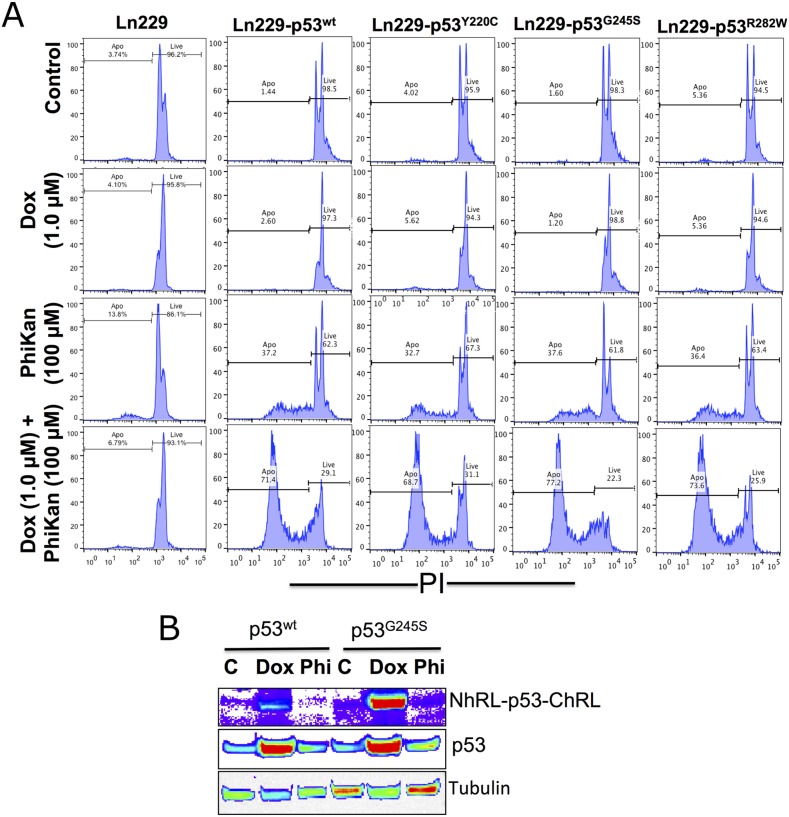
Therapeutic response of parental Ln229 cells and Ln229 cells stably expressing split-*Renilla* luciferase complementation biosensor with different p53 mutants (p53^wt^, p53^Y220C^, p53^G245S^, p53^R282W^) in response to Doxorubicin (Dox) alone or in combination with PhiKan083, and its associated p53 protein stabilization in cells **(A)** Cells analyzed for apoptosis population 24 h after treatment with Dox (1 μM) alone or in combination with PhiKan083 (100 μM) by PI staining based FACS analysis. **(B)** Immunoblot analysis for measuring the change in endogenous p53 protein and p53-biosensor protein levels.

We tested all variants of Ln229 cells (p53^wt^, p53^Y220C^, p53^G245S^, p53^R282W^), along with parental Ln229 cells, for their responses consequent to changes in their endogenous p53 protein type. For this, we treated the cells with Dox alone or in combination with PhiKan083, and measured p53 protein stabilization and the associated therapeutic response. Apoptosis in cell populations 24 h after treatment with Dox alone (1 μM) or Dox in combination with PhiKan083 (Dox 1 μM plus PhiKan083 100 μM) showed significantly increased levels of apoptosis in all cell variants following combination treatment compared to controls, or Dox or PhiKan083 alone as monotherapies, while the response was absent or very poor in parental Ln229 cells (Figure 5A). Treatment using Dox alone resulted in no significant change in the levels of apoptotic cells, while PhiKan083 alone induced nearly 30% apoptosis. In contrast, when Dox (1 μM) was combined with PhiKan083 (100 μM), there was significant enhancement in apoptosis in all cell variants. Dox is reported to enhance endogenous p53 protein through stabilization. Hence, we tested all variant cells to measure the levels of endogenous p53 protein plus the transduced biosensor fusion protein, using immunoblot analysis. We found significant enhancement in the levels of both endogenous p53 protein and the biosensor fusion protein in cells treated with Dox alone, but it was not observed in either PhiKan083 alone or PhiKan083 in combination with Dox (Figure 5B).

### Treatment of cells with PhiKan083 and SCH529074 showed a significant synergistic effect when combined with TMZ or Dox

**Figure 6:**
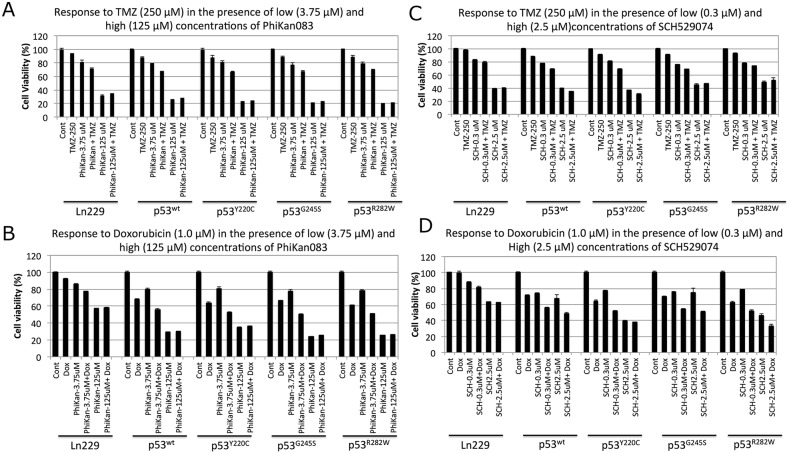
Therapeutic response of Ln229 cells stably expressing split-*Renilla* luciferase complementation biosensor with different p53 variants (p53^wt^, p53^Y220C^, p53^G245S^, p53^R282W^) to chemotherapies in the presence of low and high doses of PhiKan083 and SCH529074 by MTT assay **(A)** Tezmozolomide (250 μM) in the presence of low (3.75 μM) and high (125 μM) dose of PhiKan083; **(B)** Doxorubicin (1 μM) in the presence of low (3.75 μM) and high (125 μM) dose of PhiKan083; **(C)** Tezmozolomide (250 μM) in the presence of low (0.3 μM) and high (2.5 μM) dose of SCH529074; **(D)** Doxorubicin (1 μM) in the presence of low (0.3 μM) and high (2.5 μM) dose of SCH529074.

The ultimate aim for the use of drugs that induce p53 refolding is to sensitize cancer cells to low concentrations of clinically used chemotherapeutic drugs by reviving p53-tumor-suppressor-mediated apoptotic pathways. The RBA of PhiKan083 is ~150 μM, while that for SCH529074 is ~5.0 μM. These concentrations are considerably toxic to cancer cells, which would limit their use for *in vivo* applications owing to the consequent unintended toxicity to normal cells as well. We therefore tested drug dosages that differed from these RBA values for their potential use in combination treatments with current chemotherapies. We used all five p53 variants of Ln229 cells with 250 μM TMZ or 1 μM Dox, in combination with different concentrations of PhiKan083 (0.375 μM to 250 μM) and SCH529074 (0.3 μM to 20 μM). We tested the cells for their viability 48 h after treatment by an MTT assay. We found a significant synergistic effect by both Dox and TMZ when we used with low concentrations of PhiKan083 (up to 31.25 μM) and SCH529074 (up to 1.25 μM). In contrast the high concentrations of these drugs induced maximum levels of cell death without the supplementation of either Dox or TMZ (Supplementary Figure 4, and Figure 6). Of note, we observed an enhanced synergistic effect from variants of cells stably expressing various p53 biosensors when compared to parental Ln229 cells. In addition, Dox showed an enhanced synergistic effect when compared to TMZ in cells with all different p53 variants. This synergistic effect was significantly higher in cells co-treated with SCH529074 and Dox when compared to PhiKan083 in combination with Dox. Improving the RBA of both SCH529074 and PhiKan083 by conducting structural activity relationship (SAR) analysis to synthesize similar drugs with high affinity for various p53 mutants, or identifying drugs that show high affinity binding to selective mutants that enhance refolding and reactivation of p53 protein function would improve the synergistic effect of chemotherapy.

### Evaluation of cells expressing different p53 variant biosensors for their treatment response to PhiKan083 at different doses, in combination with various concentrations of TMZ, showed a significantly altered synergistic effect selectively by each variant and different from parental Ln229 cells

**Figure 7:**
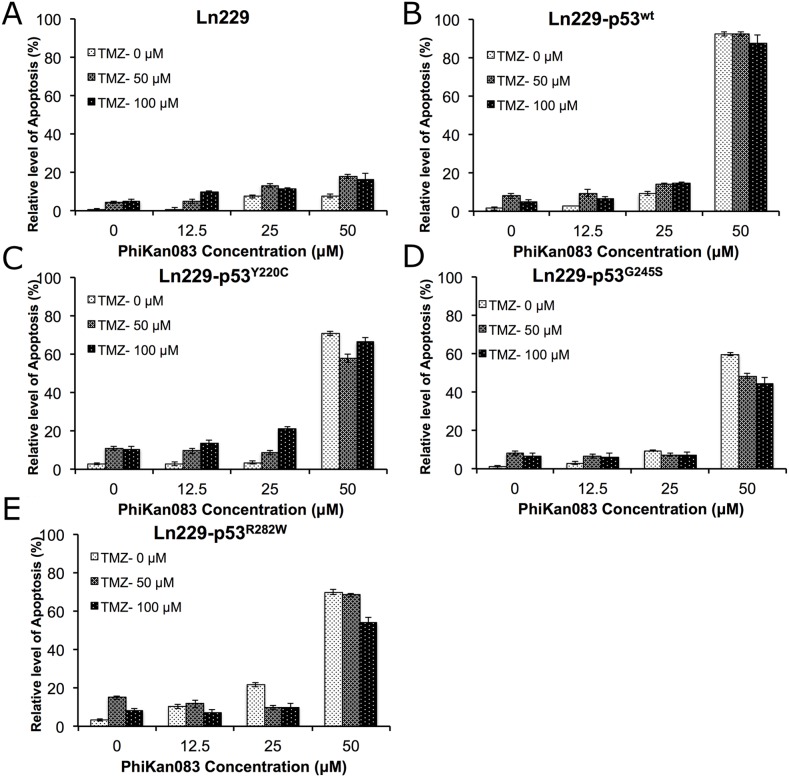
Differential therapeutic response of Ln229 cells stably expressing split-*Renilla* luciferase complementation biosensor with different p53 mutants (p53^wt^, p53^Y220C^, p53^G245S^, p53^R282W^) to chemotherapy (TMZ) in the presence of different doses of PhiKan083, as assessed by FACS analysis for the induced apoptotic population Graph showing the quantitative results of parental Ln229 cells **(A)** and, Ln229 variant cells stably expressing different p53 biosensors: Ln229-NRluc-p53^wt^-CRluc **(B)**, Ln229-NRluc-p53^Y220C^-CRluc **(C)**, Ln229-NRluc-p53^G245S^-CRluc **(D)**, Ln229-NRluc-p53^R282W^-CRluc **(E)**, tested in response to the different treatment doses of PhiKan083 (0, 12.5, 25 and 50 μM) or Tezmozolomide (0, 50 and 100 μM), and as a combination.

Since we observed a synergistic treatment effect when we combined a low concentration of PhiKan083 (3.75 μM) with either TMZ (250 μM) or Dox (1 μM) in Ln229 cells expressing different p53 biosensor variants, we further evaluated the altered therapeutic response by these variants to PhiKan083 (0-100 μM) in combination with different doses of TMZ (0-100 μM). Instead of an MTT assay to measure cell viability alone, we used propidium iodide (PI) staining based FACS analysis to accurately quantify the number of live and apoptotic cell populations. We analyzed samples 48 h after different treatment conditions. We found significantly increased numbers of apoptotic cells in Ln229 cells stably expressing different p53 biosensor variants compared to parental Ln229 cells, with the highest efficiency in cells expressing the p53^Y220C^ variant (apoptotic populations in cells with treatment condition at 100 μM TMZ and 50 μM PhiKan083 were – Ln229 cells: 16.2%; Ln229-p53^wt^: 87.6%; Ln229-p53^Y220C^: 66.3%; Ln229-p53^G245S^: 44.3%; and Ln229-p53^R282W^: 53.9%) (Figure 7, and Supplementary Figure 5).

### Treatment of cells with SCH529074 at different doses in combination with various concentrations of TMZ showed a significantly enhanced apoptotic effect in all Ln229 variants

**Figure 8:**
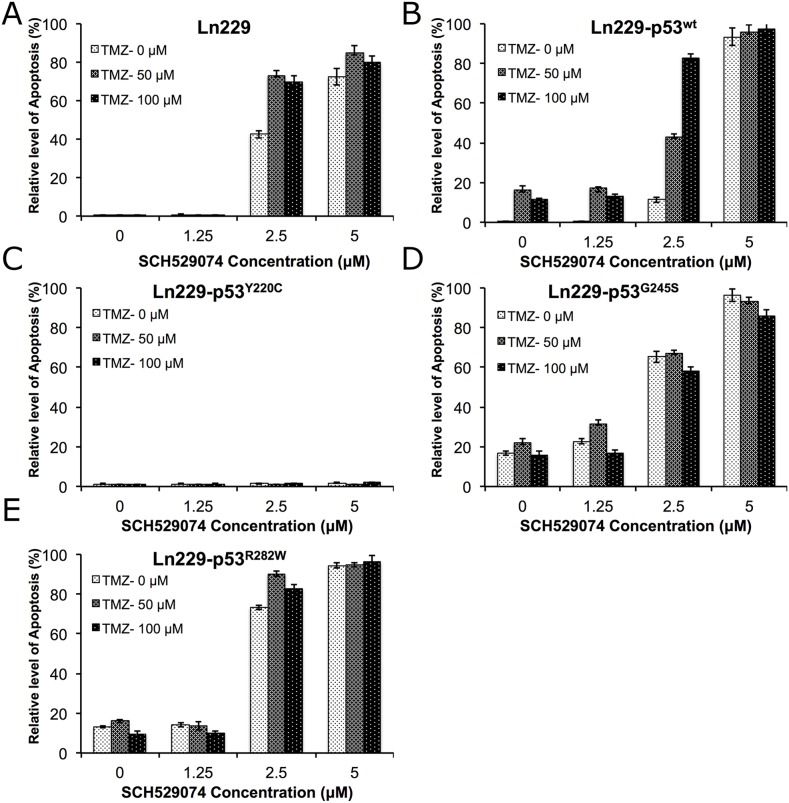
Differential therapeutic response of Ln229 cells stably expressing split-*Renilla* luciferase complementation biosensor with different p53 variants (p53^wt^, p53^Y220C^, p53^G245S^, p53^R282W^) to chemotherapy (TMZ) in the presence of different doses of SCH529074 as assessed by FACS analysis for the induced apoptotic population Graph showing the quantitative results of parental Ln229 cells **(A)** and, Ln229 variant cells stably expressing different p53 biosensors: Ln229-NRluc-p53^wt^-CRluc **(B)**, Ln229-NRluc-p53^Y220C^-CRluc **(C)**, Ln229-NRluc-p53^G245S^-CRluc **(D)**, Ln229-NRluc-p53^R282W^-CRluc **(E)**, tested in response to the different treatment doses of SCH529074 (0, 1.25, 2.5 and 5.0 μM) or Tezmozolomide (0, 50 and 100 μM) and as combination.

In addition to studying the dose dependent response of PhiKan083 in combination with TMZ, we also evaluated the dose dependent effect of SCH529074 (0-5.0 μM) in combination with different concentrations of TMZ (0-100 μM) to determine potential enhancement of therapeutic response in Ln229 cells and their p53 variants. We used similar treatment conditions and their analysis as described above. We found a significantly increased number of apoptotic cells in parental Ln229 cells treated with SCH529074, in contrast to PhiKan083 treatments where cells expressing different p53 variants showed the enhanced apoptosis (apoptotic populations in cells with treatment condition at 100 μM TMZ and 2.5 μM SCH529074 were – Ln229 cells: 69.4%; Ln229-p53^wt^: 82.9%; Ln229-p53^Y220C^: 0.46%; Ln229-p53^G245S^: 56.9% Ln229-p53^R282W^: 82.6%) (Figure 8, and Supplementary Figure 6). Of interest, we observed significant resistance to treatment by Ln229 cells expressing the p53^Y220C^ variant.

### Immunoblot analysis further supported the differential response of cells to a combination treatment using Dox and TMZ with PhiKan083 and SCH529074

**Figure 9:**
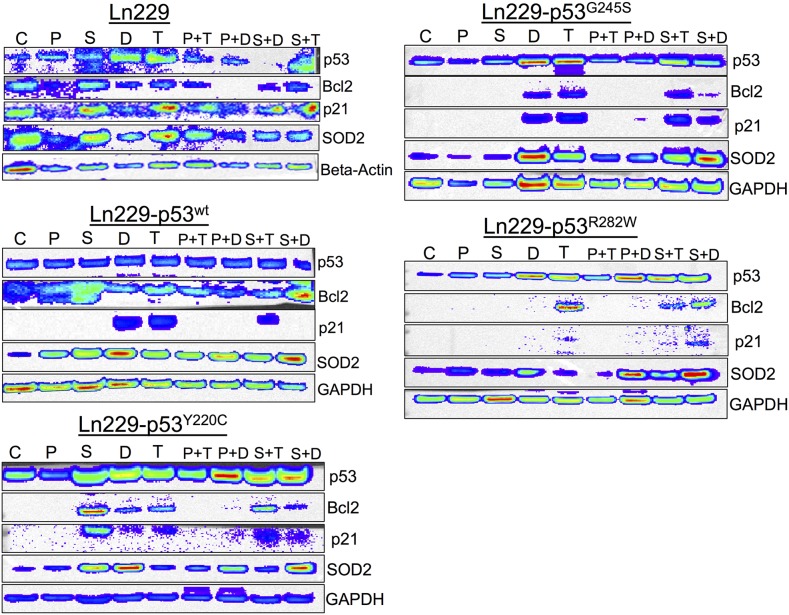
Differential expression of p53 responsive pathway proteins (p21, Bcl2 and SOD2) in response to the treatment of PhiKan083 and SCH529074 in combination with Dox and TMZ (C: Control, P: PhiKan083-50 μM, S: SCH529074-1. 25 μM, D: Dox-1 μM, T: TMZ-100 μM, P+D: PhiKan083-50 μM and Dox-1 μM, P+T: PhiKan083-50 μM and TMZ-100 μM, S+D: SCH529074-1.25 μM and Dox-1 μM, S+T: SCH529074-1.25 μM and TMZ-100 μM)

We tested cells treated with PhiKan083 and SCH529074 in combination with Dox and TMZ for their differential response by scrutinizing treatment impact on important pathway proteins. We focused on the direct targets of p53, such as p21 (cytostatic response) and Bcl2 (apoptotic response), and other related pathway proteins such as SOD2 (antioxidant response). We found significantly altered expression of these proteins in response to treatment while respective housekeeping genes were expressed at normal levels (Figure 9). We found that p53 protein was stabilized in all engineered variants compared to parental Ln229 cells. Bcl2 levels were significantly upregulated when cells with p53 variants were treated with SCH529074 or SCH529074 in combination with Dox, along with a concomitant downregulation of p21 levels (except in Ln229-p53^G245S^ mutant cells). In contrast, Bcl2 levels was high in cells with mutant p53^Y220C^ treated with SCH529074 but became downregulated when combined with Doxorubicin. Interestingly the cells with mutant p53^R282W^ showed prominent activation of Bcl2 expression when treated with TMZ in comparison to other variants, including parental Ln229 cells.

### SCH529074 in combination with Dox or TMZ showed enhanced therapeutic effect in U87MG cells, which is endogenously wild-type for p53 expression

In addition to evaluating small molecule drugs that bind to p53 protein and altering its cellular function in Ln229 and its p53-mutant variants, we also tested SCH529074 effects in U87MG cells, which are endogenously wild-type for p53 protein expression, by combing with either Dox or TMZ. The results showed significantly enhanced synergistic apoptotic effect for both Dox and TMZ when combined with SCH529074, compared to each drug treated independently (Supplementary Figure 7-9).

## DISCUSSION

We developed a molecular imaging biosensor that can detect and image small molecule drug-mediated refolding of mutant p53 protein into its wild-type conformation, and the consequent recovery of functional activity of p53 in cells. We used our previously developed split-reporter protein complementation systems to conceive the engineering of this new biosensor [11, 16–18]. We identified and optimized this complementation system by screening various split-RLUC and split-FLUC complementation biosensors constructed to express fusion proteins containing different split reporter protein fragment combinations. This approach identified a complementation biosensor made of fragments generated by splitting RLUC at amino acid position 229 (NRLUC: amino acids 1-229 and CRLUC: amino acids 230-311). This biosensor exhibits minimal background signal and maximum complementation efficiency when used to identify drugs that were previously reported to bind efficiently with p53 protein [18].

Regarding our choice of cell line, we engineered Ln229 glioblastoma cells that were endogenously mutant at the proline rich N-terminal domain (p53^P98L^, i.e. not affecting the DNA-binding domain), to express the biosensor containing one of four different p53 proteins: p53^wt^, p53^Y220C^, p53^G245S^ and p53^R282W^. Of note, there are several available non-glioblastoma cancer cell lines that are p53-null, e.g. Saos-2, HL-60, or H1299, but these cells would not be relevant to our line of enquiry regarding treatment of glioblastoma. In theory it would have been ideal to use a p53-null glioblastoma cell line, e.g. Ln308, for our investigation but, unfortunately, Ln308 cells are the only known p53-null glioblastoma cells [19], and they are not available freely or commercially. Moreover, it was not possible to use glioblastoma cells that were already p53-wild-type to test the biosensor. In essence the wild-type p53 phenotypic background would mask detection of any downstream effects from the induced properly re-folded p53 (upon addition of small molecule drugs) that was originally expressed as mutant unfolded p53 from the biosensor.

We used PhiKan083 and SCH529074 as p53 binders, and RITA, a small molecule drug that prevents p53 interaction with HDM2, as a negative control [20–22]. As expected, we demonstrated induction of luciferase complementation only by PhiKan083 and SCH529074, while RITA showed similar complementation levels to controls. We found that p53 with a mutation at Y220C was responsive to PhiKan083 while showing no change in complementation signal either to SCH529074 or RITA (Figure 2B). Interestingly, we observed significant activation of complementation signal from biosensors carrying wild-type p53 with use of PhiKan083 or SCH529074 (Figure 2A). This result for the first time explains that the binding of these drugs to p53 protein not only induces structural changes to mutant p53 protein, but it also can result in change in wild-type p53 protein, thus altering the distance between the -NH2 and -COOH terminals of the protein and which, in turn, facilitates the reporter protein fragment complementation observed herein.

PhiKan083 and SCH529074 are small molecule drugs that were previously reported to have relative binding affinities (Kd) to p53 protein of 150 μM and 2.5 μM, respectively [21, 23]. These drugs are highly toxic to cells at these concentrations. Using *in vitro* spectroscopic analyses, both PhiKan083 and SCH529074 were previously screened for estimating their RBAs and for their ability to induce structural changes to p53 protein. Purified p53 protein was invariably used for those studies [24]. In contrast, the assay we describe uses a biosensor fusion protein expressed in cells to measure structural changes induced in p53 protein by these drugs in live cells. Consequently, toxicity of these drugs to cells is a major problem when studying drug-induced p53 protein folding in intact cells, as well as intact cells when attempted within living experimental animals. Demonstrating the utility of newly discovered drugs that induce reactivation of mutant p53 into a wild-type conformation, and the associated functional recovery of this tumor suppressor protein in cancer cells, would be necessary for the clinical translation of these drugs to treat cancers, which, in general, possess compromised p53 mediated apoptotic signaling. Hence, identifying drugs with high binding affinity for mutant p53 proteins, especially at nanomolar concentrations, would be highly desirable in attempts to improve current clinical chemotherapeutic regimens for many cancers, while overcoming non-specific toxicity.

In addition to developing biosensors that measure the structural changes induced by small molecule drugs to p53 protein, we also tested whether the chimeric p53 protein (expressed as a fusion protein with the split-reporter protein fragments) maintains its functional property. This is because each expressed p53 monomer should be physically unimpeded in subsequently forming a functional tetramer, for it then to accomplish its downstream transcriptional activation in cells [25]. We therefore generated Ln229 cells stably expressing fusion protein biosensors containing different p53 variants (Ln229-NRluc-p53^wt^-CRluc, Ln229-NRluc-p53^Y220C^-CRluc, Ln229-NRluc-p53^G245S^-CRluc, and Ln229-NRluc-p53^R282W^-CRluc), a reflection on mutations that are frequently found in glioblastoma (Y220C: ~3%, G245S: ~7%, and R282W: ~4%) [26]. We tested these variant cells for their response to chemotherapeutic agents (Dox and TMZ) in the presence of PhiKan083 and SCH529074. Our results clearly identify differential responses of these cells to combination chemotherapy, which is entirely different from the behavior of parental Ln229 cells. This property demonstrates the viability of functional p53 proteins expressed from within the biosensor fusion proteins in cells (Figure 7 & 8). We additionally identify a cumulative therapeutic effect from Dox and TMZ when combined with PhiKan083 and SCH529074 at lower concentrations. By contrast, there was no perceptible difference when these drugs were used at high concentrations owing to their dose-associated toxicities (Figure 6-8).

## MATERIALS AND METHODS

### Ethics statement

Investigations have been conducted in accordance with the ethical standards according to the Declaration of Helsinki, according to national and international guidelines, and have been approved by the authors’ institutional review board.

### Cells, chemicals, enzymes, and reagents

We purchased human embryonic kidney (HEK293T) cells, and Ln229 and U87MG glioblastoma cells from ATCC (Manassas, VA, USA) and used these within six months of purchase. ATCC's cell line authentication and characterization tests include checking for morphology using microscopy, growth curve analysis, isoenzymology for species verification, DNA fingerprinting for identity verification of human cell lines, and mycoplasma detection. We obtained cell culture medium, FBS, penicillin, streptomycin, sodium bicarbonate, and all cell culture plates from GIBCO BRL (Frederick, MD, USA). We purchased Lipofectamine 2000 transfection reagent from Invitrogen (Carlsbad, CA. USA), and all restriction and modification enzymes and ligase from New England Biolabs (Beverly, MA, USA). We used the nucleotide fragments for various plasmids and lentiviral constructions to create p53-folding biosensors with different luciferases (FLUC and RLUC) using our plasmid bank. We used plasmid extraction kits and DNA gel elution kits from Qiagen (Valencia, CA, USA) an d Epoch Life Sciences (Missouri City, TX, USA). We purchased D-luciferin from Biosynth (Switzerland), coelenterazine from Nanolight (Pinetop, AZ, USA), antibiotics for bacterial and cell culture experiments from Sigma (St. Louis, MO, USA), bacterial culture media from Difco (Franklin Lakes, NJ, USA), and Taq DNA-polymerase for PCR amplification from 5-Prime (Gaithersburg, MD, USA). Primers for PCR amplifications were synthesized at the Stanford Protein and Nucleic Acid (PAN) Facility (PAN, Stanford). Sequencing of plasmid vectors were carried out by Sequetech DNA sequencing service (Mountain View, CA, USA). We purchased the p53 activator, SCH529074, from Cayman (Ann Arbor, Michigan, USA), PhiKan083, Dox and TMZ from Tocris Biosciences (Minneapolis, MN, USA), RITA from Cayman (Ann Arbor, MI, USA), antibodies for p53, β-actin, Bcl2, SOD2 and p21 from Cell Signaling Technology (Beverly, MA, USA), and GAPDH and tubulin primary antibodies from Santa Cruz Biotechnology (B-5 −1-2, Santa Cruz Biotechnology, Santa Cruz, CA, USA).

### Construction of plasmid and lentiviral vectors expressing p53 protein folding biosensors

We constructed different p53 protein folding biosensors flanking N- and C-terminal luciferase fragments (FLUC and RLUC) using a standard PCR based cloning strategy. In brief, we first constructed wt-p53 protein sandwiched between N- and C-terminal luciferase fragments of both NFLUC and CFLUC or NRLUC and CRLUC with different split sites in a pcDNA 3.1 (+) vector backbone as follows: NheI-NFLUC/NRLUC-BamHI-Linker-wt-p53-Linker-BamHI-CFLUC/CRLUC. We used split-luciferase fragments of amino acids 1-398 (NFLUC) and 394-550 (CFLUC) for the Firefly luciferase complementation system, and fragments generated at amino acid position 110 (NRLUC: 1-110 and CRLUC: 110-311) and 229 (NRLUC: 1-229 and CRLUC: 230-311) for the *Renilla* luciferase complementation system. We used site directed mutagenesis to create a single amino acid change at position 220 (p53^Y220C^), 245 (p53^G245S^) and 282 (p53^R282W^) using standard forward and reverse primers with inserted mutant nucleotides. We then further used the sequence confirmed vector constructs in cell culture transient transfection experiments and for sub-cloning into a lentiviral backbone. The full-length fusion fragments of NRLUC-p53-CRLUC, based on an RLUC complementation system of split-site 229, were further released along with the constitutive Ubiquitin promoter (Ubiquitin-NRLUC-p53-CRLUC) using SpeI and PmeI restriction enzyme sites and sub-cloned into the respective enzyme digested lentiviral backbone (pHAGE-Ubi-dTomato). We then chose the sequence confirmed vectors for lentiviral production using the three-vector transfection system described below.

### Transient transfection, drug treatment and bioluminescence assay

To compare luciferase activity from various engineered complementation biosensors, we transiently transfected HEK293T and U87MG cells with respective vectors and treated with PhiKan083, or SCH529074, or RITA (a negative control, see Discussion) at concentrations based on the relative binding affinity (RBA) of respective mutants, and then assessed for luciferase activity using an IVIS optical imaging system (Perkin Elmer, Bridgeville, PA, USA). In brief, we transfected cells in 12-well culture plates (at concentrations of 1.5 × 10^5^ cells/well) with 1.5 μg/well of respective plasmid using Lipofectamine 2000 transfection reagent, as per the manufacturer's protocol. After 24 h we washed cells once with PBS and treated with 50 μM of PhiKan083 for 16 h, and then imaged for luciferase activity by the addition of D-luciferin (50 μg/ml) or coelenterazine (10 μg/ml) using the IVIS-optical imaging system. We quantified luciferase activity using Living Image quantitation software (Perkin Elmer, Bridgeville, PA, USA). After imaging we lysed the cells, quantified the protein concentrations, and normalized the results.

### Cell culture, lentiviral production, and stable cell generation by lentiviral transduction

We maintained HEK293T, U87MG and Ln229 cells in Dulbecco's Modified Eagle's Medium (11995, Life Technologies) supplemented with 10% fetal bovine serum, 100 U/ml penicillin and 0.1% streptomycin. We performed transient transfections on 80% confluent cells using the lipofectamine 2000 transfection reagent (Life Technologies). 1.5 μl of lipofectamine 2000 was used for every μg of plasmid DNA. Plasmid DNA-lipid complexes were prepared in serum free Opti-MEM Medium (Life Technologies, Carlsbad, CA, USA). For lentiviral production we used HEK293T and Ln229 cells grown in Dulbecco's Modified Eagle's Medium supplemented with 10% FBS, 100 U/ml penicillin and 0.1% streptomycin. We used a three-vector transfection system (pHAGE + VPR + VSVG) to produce lentivirus for split-RLUC complementation biosensors expressing p53^wt^, p53^Y220C^, p53^G245S^ and p53^R282W^. HEK293T cells cultured in DMEM supplemented with 10% FBS, 100 U/ml penicillin and 0.1% streptomycin were transfected with all three plasmids (pHAGE + VPR + VSVG) in a 3:2:1 ratio using a standard calcium phosphate transfection method. Twenty four hours later we washed the cells once with PBS and replaced with 7.5 ml of complete medium containing 10 mM Hepes buffer. We enriched virus from the medium 48 h after medium change by ultracentrifugation. After titration we used viruses for making Ln229 cells stably expressing all four biosensors. In brief, we transduced the cells plated to 80% confluence in 10 cm plates 24 h before transduction. We washed the cells once with PBS, and then added 2 ml of 1 × 10^8^ PFU virus mixed with 3 ml of serum free Opti-MEM and 5 μl of 1 mg/ml Polybrene. Each plate was incubated for 4 h at 37°C and 5% CO_2_ with intermittent mixing. Four hours later we washed the plate once with PBS and supplemented with 5 ml of complete medium containing 10% FBS, 100 U/ml penicillin and 0.1% streptomycin. We sub-cultured the cells twice before we FACS sorted them to enrich a population of cells having equal numbers of inserts using the co-expressed dTomato marker. The enriched cells were used for further experiments.

### Immunoblot analysis

Six million cells of each batch of Ln229 cells stably expressing NRLUC-p53-CRLUC constructs were harvested by trypsinization, spun at 5,000 rpm for 5 min, and the cell pellets were lysed in RIPA buffer containing protease inhibitor cocktail and EDTA by sonication thrice at 40% amplitude for 15 s each. We centrifuged the cell lysates at 15,000 rpm for 15 min at 4°C to remove any insoluble membrane proteins before they were used for protein estimation using the Nanodrop. We prepared protein samples, containing 200 μg of total protein in 1X Lamelli loading buffer with 5% β-mercaptoethanol (Life Technologies, Carlsbad, CA, USA), which were denatured at 95°C for 5 min, resolved on 4-12% SDS-polyacrylamide pre-cast gels (Life Technologies, Carlsbad, CA, USA) and electroblotted onto a polyvinylidene difluoride nylon membrane (Bio-Rad, Hercules, CA, USA). We used the SeeBlue Pre-Stained Standard (Life Technologies, Carlsbad, CA, USA) to enable molecular weight estimations. Membranes were blocked in 5% non-fat dry milk in Tris-buffered saline containing 0.01% Tween-20 (TBST) followed by incubation with a primar y antibody. As appropriate, we used antibodies suitable for the detection of p53 (#9282, Cell Signaling, Beverly, MA, USA). Following three TBST washes, we incubated membranes with the appropriate horseradish-peroxidase conjugated secondary antibody (Sigma Aldrich, St Louis, MI, USA). After three additional TBST washes, we incubated immunoblots with the Pierce ECL Western Blotting Substrate (Thermo Scientific, Waltham, MA, USA) for 1 min, and then used the IVIS-Lumina imaging system (Perkin Elmer, Bridgeville, PA, USA) to detect and measure chemiluminescent signals and bands respectively. A GAPDH or tubulin or ?-actin primary antibody was used as a loading control antibody. Similarly, for measuring change in response to treatment in the biosensor proteins, we used samples from Ln229 cells stably expressing p53 biosensors (NRLUC-p53-CRLUC) after treating with SCH529074 and PhiKan083.

### Cell imaging assays to measure drug modulated p53 folding-assisted complementation signal in Ln229 cells stably expressing the biosensors

Ln229 cells stably expressing split-RLUC complementation biosensors with different p53 proteins were treated with PhiKan083 or SCH529074 in different doses for various treatment durations. We plated 10,000 cells/well in 96-well black walled plates 24 h before treating with drugs. We treated cells with PhiKan083 (15.6, 31.25, 62.5, 125 and 250 μM) and SCH529074 (0.625, 1.25, 2.5, 5.0 and 10 μM) for durations of 1 h to 16 h. After treatment, we washed the plates once with PBS and added 2 μg of coelenterazine in 50 μl of PBS to each well very quickly using a multi-well pipette. We imaged the plates by continuous acquisition of 1 min integration times for a total of 15 mins. We quantified luciferase signals from each well using Living Image software by drawing regions of interest (ROIs) over each well. We plotted the relative signal in response to treatment as a graph.

### Combination chemotherapy response by Ln229 cells engineered to express complementation biosensors with different p53 variants

We tested the effects of SCH529074 and PhiKan083 in combination with Dox and TMZ on Ln229 cells and their engineered variants, assessing for apoptotic effects and cell cycle status at different time points after treatment. The cells plated in 96-well (5,000 cells/well) or 12-well (75,000 cells/well) culture plates were treated with SCH529074, PhiKan083, TMZ and Dox individually or in combinations (as shown in the Results section) for different durations (24 h to 72 h), and assessed either by MTT assay (96-well) or PI staining-based FACS analysis. To perform MTT assays after each planned drug treatment duration, we washed the cells once with PBS and added MTT reagent (12.5 μM) in 25 μl of phenol red free DMEM with 10% FBS and 1% P&S. We incubated the cells in MTT solution for 2 h and aspirated the medium without disturbing Formazon crystals. We dissolved the crystals in 100 μl DMSO and measured absorbance at 540 nm. The readings were plotted and compared with controls for measuring the relative levels of cell viability after different treatment conditions. For FACS analysis, we collected cells in 12-well plates after treatment by trypsinization, including dead cells in the medium. We washed the cells once with PBS and fixed them in ice cold 70% ethanol for 2 h at minus 20°C. After fixation we collected cells by centrifugation and stained in 1 ml of PBS containing 10 μg/ml PI, 100 μg/ml RNAse A and 0.05% Triton X-100. We incubated the cells in PI solution for 15 min and washed once with PBS, re-suspended in 0.5 ml PBS, and assessed for live and dead cell counts using InCyte Software in a Guava FACS analyzer, and for cell cycle status using the Guava cell cycle analysis software (EMD Millipore). We analyzed the results using FlowJo software for measuring live/dead cells and cell cycle status.

### Determination of luciferase activity in cell lysates

We seeded HEK293T cells in triplicate in 24-well plates and transiently co-transfected with 0.5 μg of an eGFP fusion construct and 0.5 ng of the RLUC expression vector. We harvested plates 48 h after transfection and lysed each well in 100 μl 1X Passive Lysis Buffer (Promega, Madison, WI, USA) on a shaking platform for 10 min. Lysates were then centrifuged at 10,000 g for 3 min to remove cell debris. A 10 μl sample of cleared supernatant was assayed for FLUC activity by the addition of 50 μl LARII substrate (Promega, Madison, WI, USA). A second 10 μl sample of cleared supernatant was assayed for RLUC activity by the addition of 50 μl of 10 μg/ml coelenterazine (Nanolight, Pinetop, AZ, USA). Bioluminescent signal was determined using a 20/20n luminometer (Turner Biosystems, Sunnyvale, CA, USA). We used the Bio-Rad Protein Assay (Bio Rad, Hercules, CA, USA) to determine protein concentration usi ng 5 μl samples of cleared supernatant. FLUC activity was normalized for RLUC activity as well as total protein.

### Determination of dTomato expression in cells by fluorescence microscopy

Fluorescent micrographs of intact cells were captured using an Olympus 81X fluorescent microscope for dTomato expression in Ln229 cells transduced to stably express the different p53 biosensors. The signal was quantified using ImageJ software by drawing regions of interest.

### Determination of luciferase activity in live cells

We seeded HEK293T cells in triplicate in 24-well plates and transiently co-transfected with 0.5 μg of each p53 fusion construct and 0.5 ng of the FLUC expression vector. We prepared two sets of 24-well plates for luciferase activity assays. After 48 h, we removed medium and assayed one set of plates for FLUC activity by the addition of 200 μl/well D-luciferin. The second set of plates was assayed for RLUC activity by the addition of 200 μl/well of 10 μg/ml coelenterazine. We determined bioluminescent signal using the IVIS-Lumina imaging system. FLUC activity was normalized with RLUC signal.

### Statistical analysis

We performed statistical analyses and prepared graphical presentations using Prism (GraphPad). Most results were presented as mean ± SEM. Median fluorescence intensity, measured by FACS analysis, was presented as median ± MAD. Results were representative of at least three independent experiments. Statistical differences between means were determined using one-way ANOVA with Tukey's multiple comparison test. Observations with a p-value less than 0.05 were considered statistically significant.

## CONCLUSIONS

We describe a novel reporter-based PCA and molecular imaging biosensor to accurately detect and measure the structural folding changes induced by small molecule drugs in mutant p53 proteins within their native cellular environments. The developed cell-based system maintains the biological fidelity of the protein and, therefore, can predict the function of p53 more closely than available *in vitro* assays using purified protein. Moreover, the proposed assay system should facilitate rapid and high-throughput screening of drugs that can induce p53 protein folding to combat cancers mutant for the p53 tumor suppressor protein. The molecular imaging methodology described herein could be easily extrapolated to non-invasive, living subject studies of protein folding in various small animal models by using optical bioluminescence imaging [9, 15]. In summary, we have developed a novel reporter-based biosensor system to screen, evaluate and characterize drugs that improve cancer therapy by reactivating p53 protein function in cancer cells.

## SUPPLEMENTARY MATERIALS FIGURES AND TABLES



## References

[1] Vendruscolo M, Zurdo J, MacPhee CE, Dobson CM (2003). Protein folding and misfolding: a paradigm of self-assembly and regulation in complex biological systems. Philos Trans A Math Phys Eng Sci.

[2] Herczenik E, Gebbink MF (2008). Molecular and cellular aspects of protein misfolding and disease. FASEB Journal.

[3] Dobson CM (2003). Protein folding and misfolding. Nature.

[4] Chaudhuri TK, Paul S (2006). Protein-misfolding diseases and chaperone-based therapeutic approaches. FEBS J.

[5] Hanahan D, Weinberg RA (2000). The hallmarks of cancer. Cell.

[6] Olivier M, Hollstein M, Hainaut P (2010). TP53 mutations in human cancers: origins, consequences, and clinical use. Cold Spring Harbor Perspectives in Biology.

[7] Arya AK, El-Fert A, Devling T, Eccles RM, Aslam MA, Rubbi CP, Vlatkovic N, Fenwick J, Lloyd BH, Sibson DR, Jones TM, Boyd MT (2010). Nutlin-3, the small-molecule inhibitor of MDM2, promotes senescence and radiosensitises laryngeal carcinoma cells harbouring wild-type p53. Br J Cancer.

[8] Bernier V, Lagace M, Bichet DG, Bouvier M (2004). Pharmacological chaperones: potential treatment for conformational diseases. Trends in Endocrinology and Metabolism.

[9] Paulmurugan R, Gambhir SS (2006). An intramolecular folding sensor for imaging estrogen receptor-ligand interactions. Proc Natl Acad Sci U S A.

[10] Paulmurugan R, Tamrazi A, Katzenellenbogen JA, Katzenellenbogen BS, Gambhir SS (2008). A human estrogen receptor (ER) alpha mutation with differential responsiveness to nonsteroidal ligands: novel approaches for studying mechanism of ER action. Mol Endocrinol.

[11] Paulmurugan R, Umezawa Y, Gambhir SS (2002). Noninvasive imaging of protein-protein interactions in living subjects by using reporter protein complementation and reconstitution strategies. Proc Natl Acad Sci U S A.

[12] Chan CT, Paulmurugan R, Gheysens OS, Kim J, Chiosis G, Gambhir SS (2008). Molecular imaging of the efficacy of heat shock protein 90 inhibitors in living subjects. Cancer Res.

[13] Chan CT, Paulmurugan R, Reeves RE, Solow-Cordero D, Gambhir SS (2009). Molecular imaging of phosphorylation events for drug development. Mol Imaging Biol.

[14] Chan CT, Reeves RE, Geller R, Yaghoubi SS, Hoehne A, Solow-Cordero DE, Chiosis G, Massoud TF, Paulmurugan R, Gambhir SS (2012). Discovery and validation of small-molecule heat-shock protein 90 inhibitors through multimodality molecular imaging in living subjects. Proc Natl Acad Sci U S A.

[15] Sheahan AV, Sekar TV, Chen K, Paulmurugan R, Massoud TF (2016). A molecular imaging biosensor detects *in vivo* protein folding and misfolding. J Mol Med (Berl).

[16] Paulmurugan R, Gambhir SS (2007). Combinatorial library screening for developing an improved split-firefly luciferase fragment-assisted complementation system for studying protein-protein interactions. Anal Chem.

[17] Paulmurugan R, Gambhir SS (2005). Firefly luciferase enzyme fragment complementation for imaging in cells and living animals. Anal Chem.

[18] Paulmurugan R, Gambhir SS (2003). Monitoring protein-protein interactions using split synthetic renilla luciferase protein-fragment-assisted complementation. Anal Chem.

[19] Ishii N, Maier D, Merlo A, Tada M, Sawamura Y, Diserens AC, Van Meir EG (1999). Frequent co-alterations of TP53, p16/CDKN2A, p14ARF, PTEN tumor suppressor genes in human glioma cell lines. Brain Pathol.

[20] Brown CJ, Lain S, Verma CS, Fersht AR, Lane DP (2009). Awakening guardian angels: drugging the p53 pathway. Nat Rev Cancer.

[21] Demma M, Maxwell E, Ramos R, Liang L, Li C, Hesk D, Rossman R, Mallams A, Doll R, Liu M, Seidel-Dugan C, Bishop WR, Dasmahapatra B (2010). SCH529074, a small molecule activator of mutant p53, which binds p53 DNA binding domain (DBD), restores growth-suppressive function to mutant p53 and interrupts HDM2-mediated ubiquitination of wild type p53. J Biol Chem.

[22] de Lange J, Ly LV, Lodder K, Verlaan-de Vries M, Teunisse AF, Jager MJ, Jochemsen AG (2012). Synergistic growth inhibition based on small-molecule p53 activation as treatment for intraocular melanoma. Oncogene.

[23] Bauer MR, Jones RN, Baud MG, Wilcken R, Boeckler FM, Fersht AR, Joerger AC, Spencer J (2016). Harnessing Fluorine-Sulfur Contacts and Multipolar Interactions for the Design of p53 Mutant Y220C Rescue Drugs. ACS Chem Biol.

[24] Joerger AC, Fersht AR (2010). The tumor suppressor p53: from structures to drug discovery. Cold Spring Harb Perspect Biol.

[25] Kearns S, Lurz R, Orlova EV, Okorokov AL (2016). Two p53 tetramers bind one consensus DNA response element. Nucleic Acids Res.

[26] Xu J, Qian J, Hu Y, Wang J, Zhou X, Chen H, Fang JY (2014). Heterogeneity of Li-Fraumeni syndrome links to unequal gain-of-function effects of p53 mutations. Sci Rep.

