# Supplementary material for: A protein folding molecular imaging biosensor monitors the effects of drugs that restore mutant p53 structure and its downstream function in glioblastoma cells
Source: Oncotarget. 2018 Apr 20;9(30):21495–511. doi: 10.18632/oncotarget.25138 (PMC5940411; doi:10.18632/oncotarget.25138)
Supplement: Supplementary file 1 [file oncotarget-09-21495-s001.pdf]

A protein folding molecular imaging biosensor monitors the effects of drugs that restore mutant p53 structure and its downstream function in glioblastoma cells

SUPPLEMENTARY MATERIALS

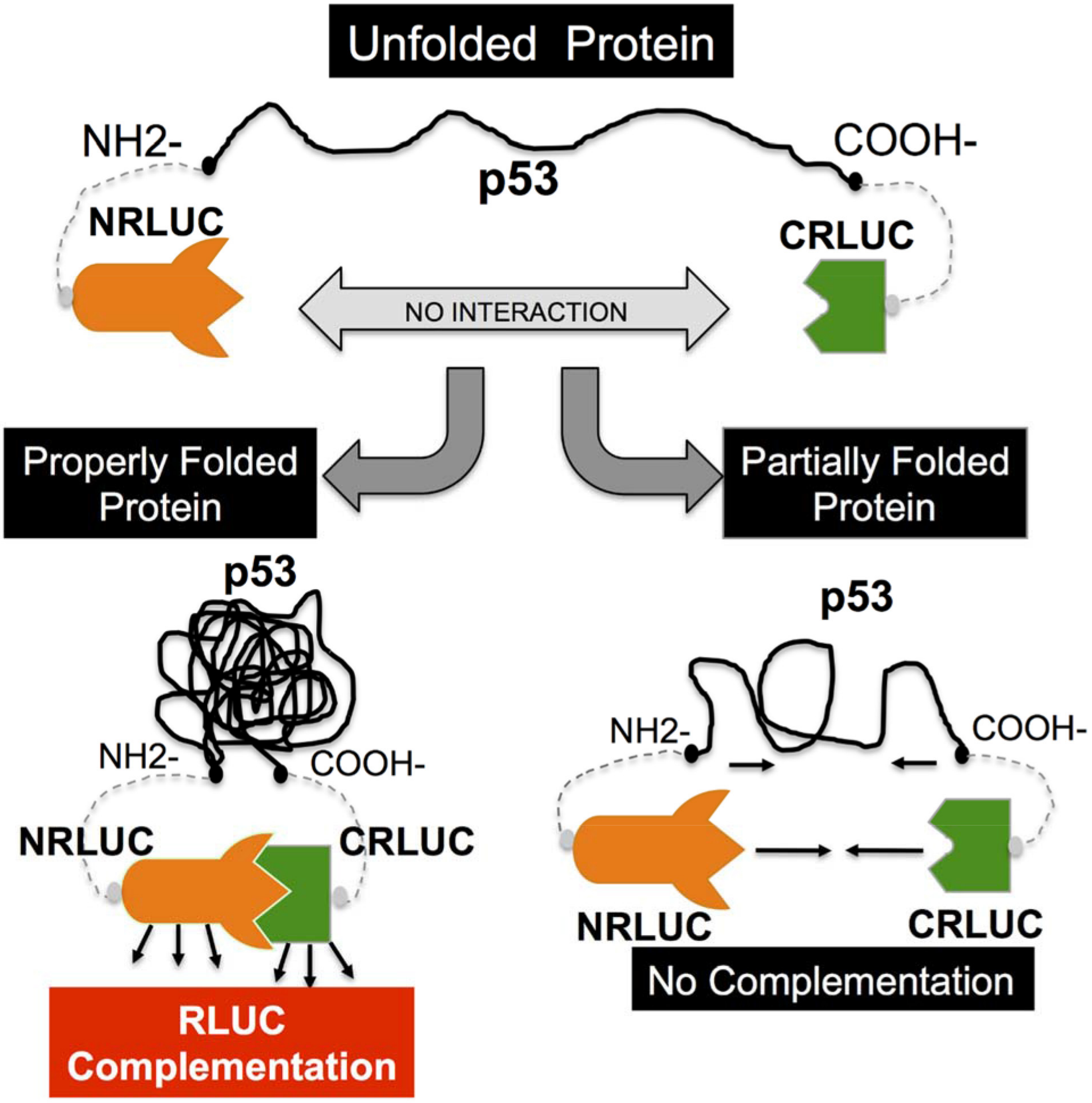

Supplementary Figure 1: Schematic figure explains functional effects of small molecule drug-induced structural changes in p53 protein measured by reporter protein complementation assay system in cells using bioluminescence imaging.

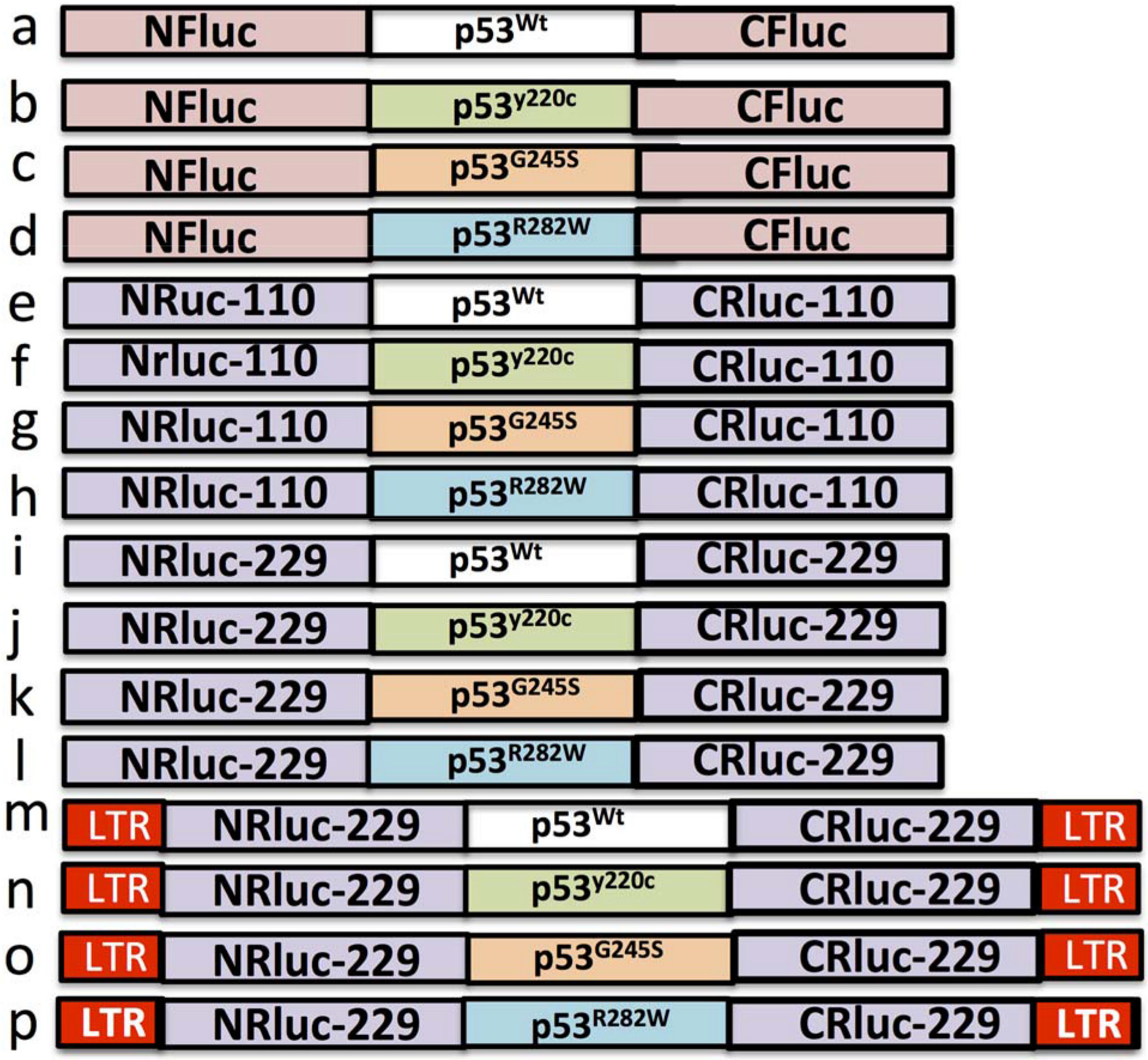

Supplementary Figure 2: Schematic figure of plasmid and lentiviral vectors constructed for studying drug-induced p53 folding in cells using reporter protein complementation system (a-p). NFLUC: N-terminal fragment of FLUC; CFLUC: C-terminal fragment of FLUC; NRLUC-110: N-terminal fragment of RLUC with split site at amino acid position 110; CRLUC-110: C-terminal fragment of RLUC with split site at amino acid position 110; N-229: N-terminal fragment of RLUC with split site at amino acid position 229; CRLUC-229: C-terminal fragment of RLUC with split site at amino acid position 229; LTR: Long tandem repeat of lentiviral vector; $p53^{wt}$: Complementation sensor constructs express wild-type p53 protein; $p53^{Y220C}$: Complementation sensor constructs express p53 protein with single amino acid mutation at amino acid position 220; $p53^{G245S}$: Complementation sensor constructs express p53 protein with single amino acid mutation at amino acid position 245; $p53^{R282W}$: Complementation sensor constructs express p53 protein with single amino acid mutation at amino acid position 282.

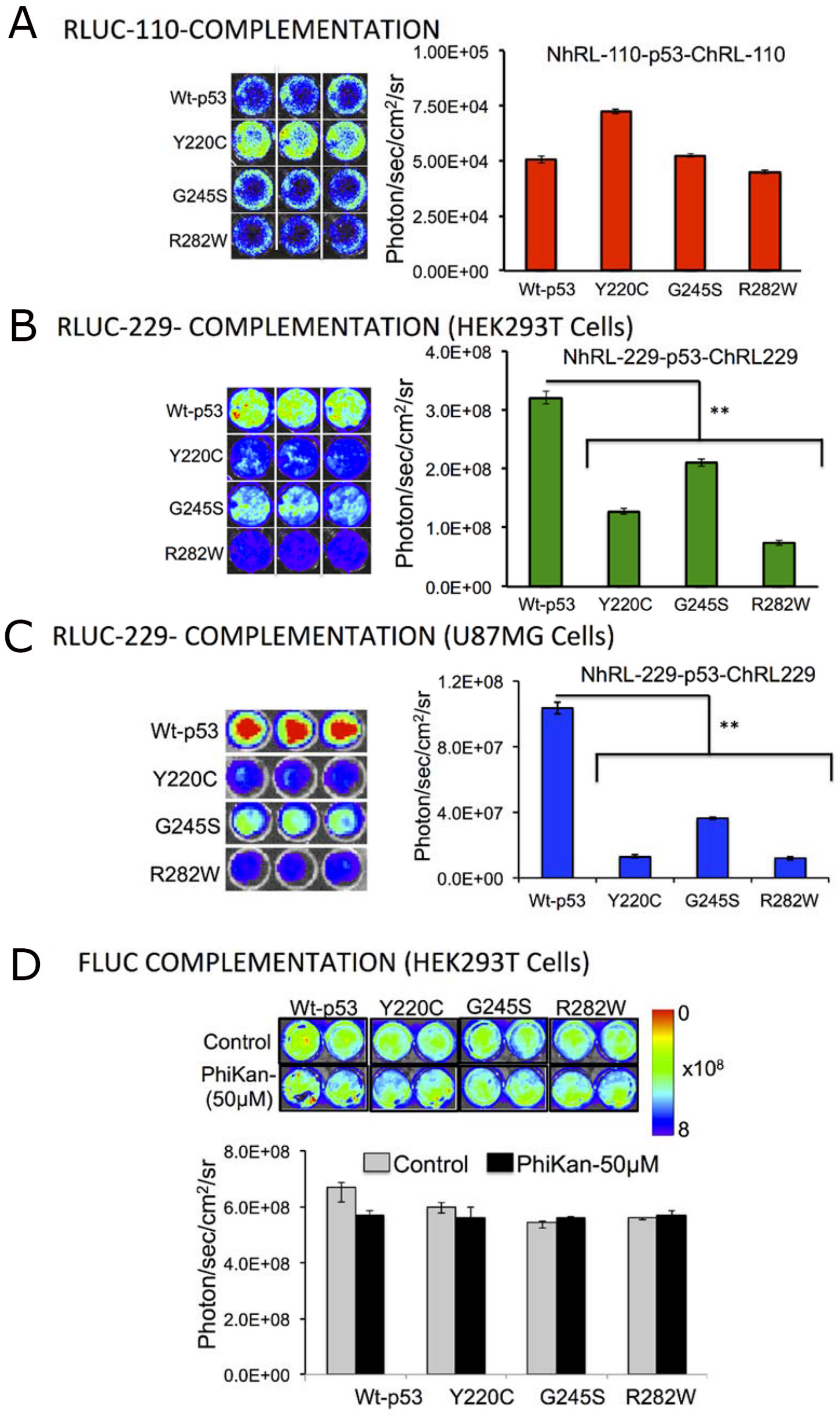

Supplementary Figure 3: Screening of split-*Renilla* and split-Firefly luciferase complementation biosensors with different fragments to identify an efficient construct that detects mutation-associated protein folding change in p53 protein. **(A)** Split-*Renilla* luciferase complementation biosensor with split fragments of amino acid at position 110 with different p53 proteins tested in HEK293 cells: Left: Optical imaging of RLUC complementation signal when using different constructs; Right: Quantitative graph of RLUC signal measured from the image. **(B-C)** Split-*Renilla* luciferase complementation sensor with split fragments of amino acid at position 229 with different p53 proteins tested in HEK293 cells (B) and U87MG cells (C): Left: Optical imaging of RLUC complementation signal when using different constructs; Right: Quantitative graph of RLUC signal measured from the image. **(D)** Split-Firefly luciferase complementation sensor with split fragments of amino acid at position 394 with different p53 proteins tested in HEK293 cells: TOP: Optical imaging of FLUC complementation signal when using different constructs; BOTTOM: Quantitative graph of FLUC signal measured from the image.

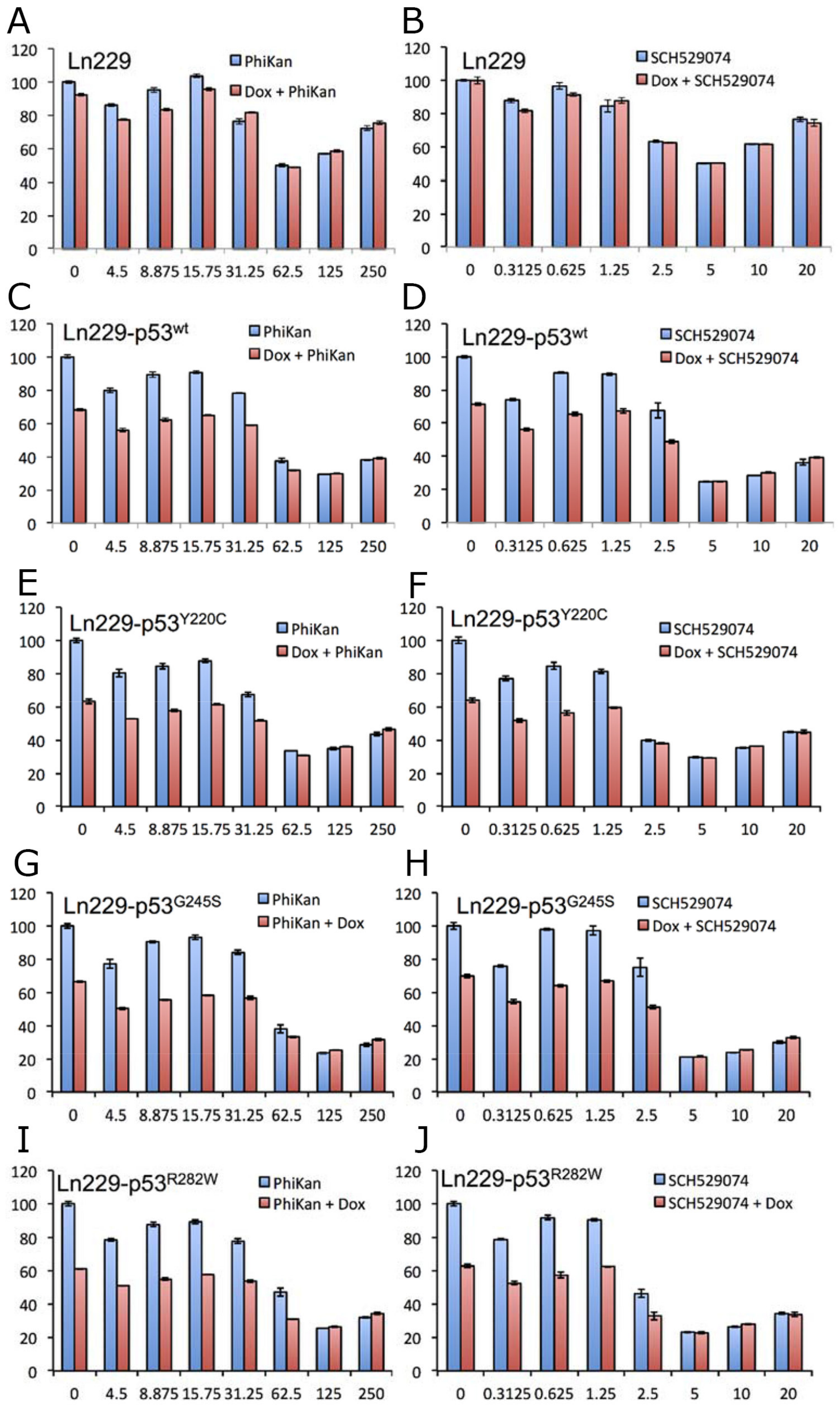

Supplementary Figure 4: (A-J). MTT assay results of Ln229 cells engineered to express different complementation biosensors and treated with PhiKan083 and SCH529074 in different concentrations, in combination with Doxorubicin (1μM), for measuring cell response to combination treatment.

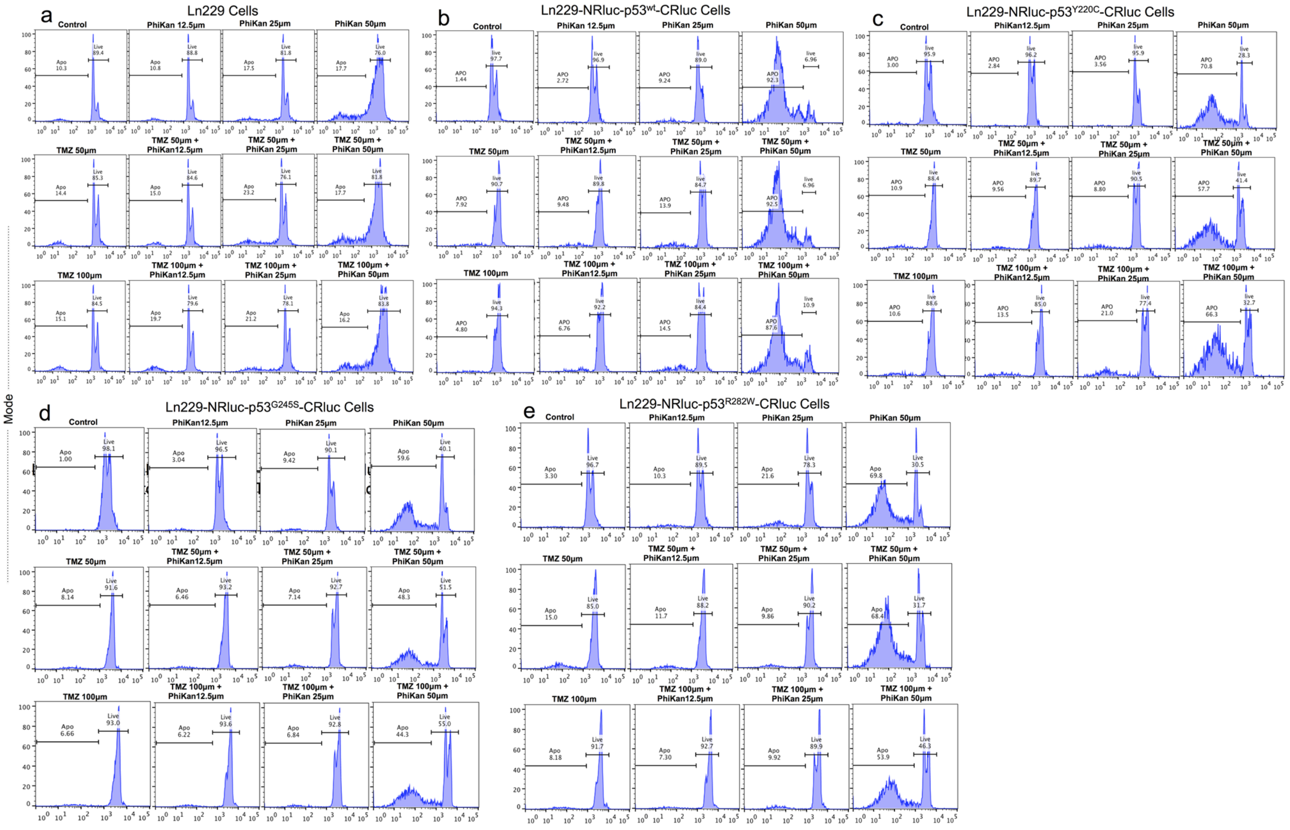

Supplementary Figure 5: FACS data reflecting the quantitative results shown in Figure 7. Differential therapeutic response of Ln229 cells stably expressing split-*Renilla* luciferase complementation biosensor with different p53 variants ($p53^{wt}$, $p53^{Y220C}$, $p53^{G245S}$, $p53^{R282W}$) to chemotherapy (TMZ) in the presence of different doses of PhiKan083, as assessed by FACS analysis for the induced apoptotic population. **(A)** Ln229 parental cells tested in response to the different treatment doses of PhiKan083 (0, 12.5, 25 and 100 µM) or Tezmozolomide (50 and 100 µM) and as a combination; **(B)** Ln229-NRluc-$p53^{wt}$-CRluc cells tested in response to the different treatment doses of PhiKan083 (0, 12.5, 25 and 100 µM) or Tezmozolomide (50 and 100 µM) and as a combination; **(C)** Ln229-NRluc-$p53^{Y220C}$-CRluc cells tested in response to the different treatment doses of PhiKan083 (0, 12.5, 25 and 100 µM) or Tezmozolomide (50 and 100 µM) and as a combination; **(D)** Ln229-NRluc-$p53^{G245S}$-CRluc cells tested in response to the different treatment doses of PhiKan083 (0, 12.5, 25 and 100 µM) or Tezmozolomide (50 and 100 µM) and as a combination; **(E)** Ln229-NRluc-$p53^{R282W}$-CRluc cells tested in response to the different treatment doses of PhiKan083 (0, 12.5, 25 and 100 µM) or Tezmozolomide (50 and 100 µM) and as a combination.

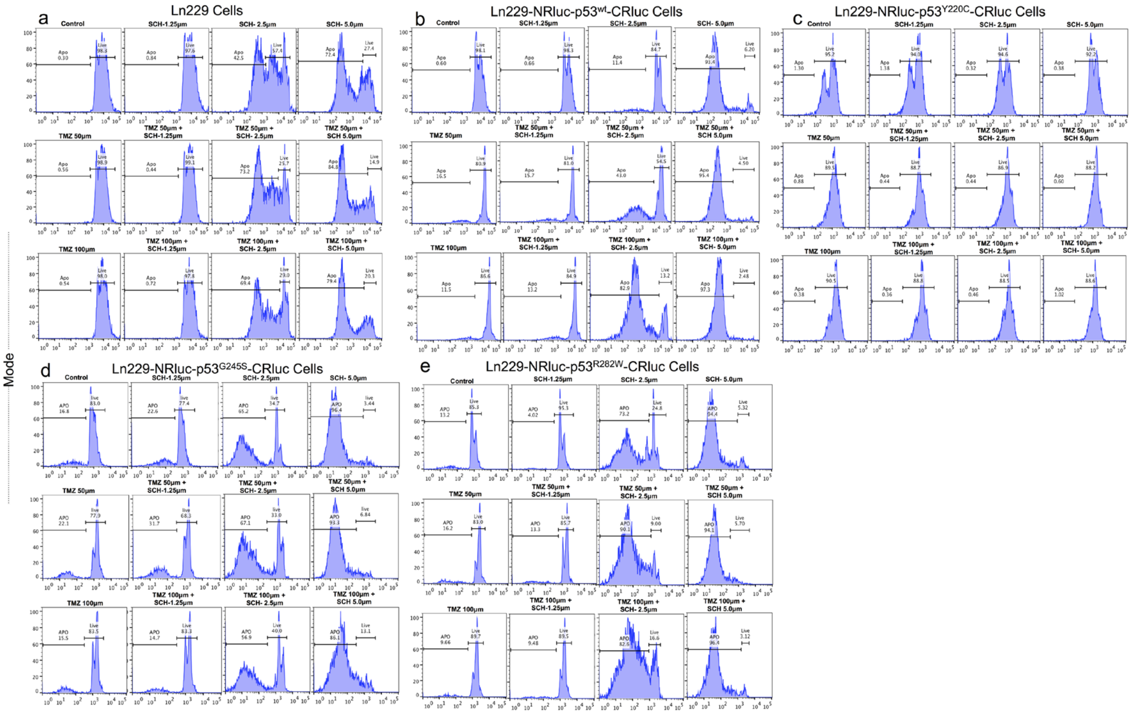

Supplementary Figure 6: FACS data reflecting the quantitative results shown in Figure 8. Differential therapeutic response of Ln229 cells stably expressing split-*Renilla* luciferase complementation biosensor with different p53 variants (p53^{wt}, p53^{Y220C}, p53^{G245S}, p53^{R282W}) to chemotherapy (TMZ) in the presence of different doses of SCH529074 as assessed by FACS analysis for the induced apoptotic population. **(A)** Ln229 parental cells tested in response to the different treatment doses of SCH529074 (0, 1.25, 2.5 and 5.0 μM) or Tezmozolomide (50 and 100 μM) and as a combination; **(B)** Ln229-NRluc-p53^{wt}-CRluc cells tested in response to the different treatment doses of SCH529074 (0, 1.25, 2.5 and 5.0 μM) or Tezmozolomide (50 and 100 μM) and as a combination; **(C)** Ln229-NRluc-p53^{Y220C}-CRluc cells tested in response to the different treatment doses of SCH529074 (0, 1.25, 2.5 and 5.0 μM) or Tezmozolomide (50 and 100 μM) and as a combination; **(D)** Ln229-NRluc-p53^{G245S}-CRluc cells tested in response to the different treatment doses of SCH529074 (0, 1.25, 2.5 and 5.0 μM) or Tezmozolomide (50 and 100 μM) and as a combination; **(E)** Ln229-NRluc-p53^{R282W}-CRluc cells tested in response to the different treatment doses of SCH529074 (0, 1.25, 2.5 and 5.0 μM) or Tezmozolomide (50 and 100 μM) and as combination.

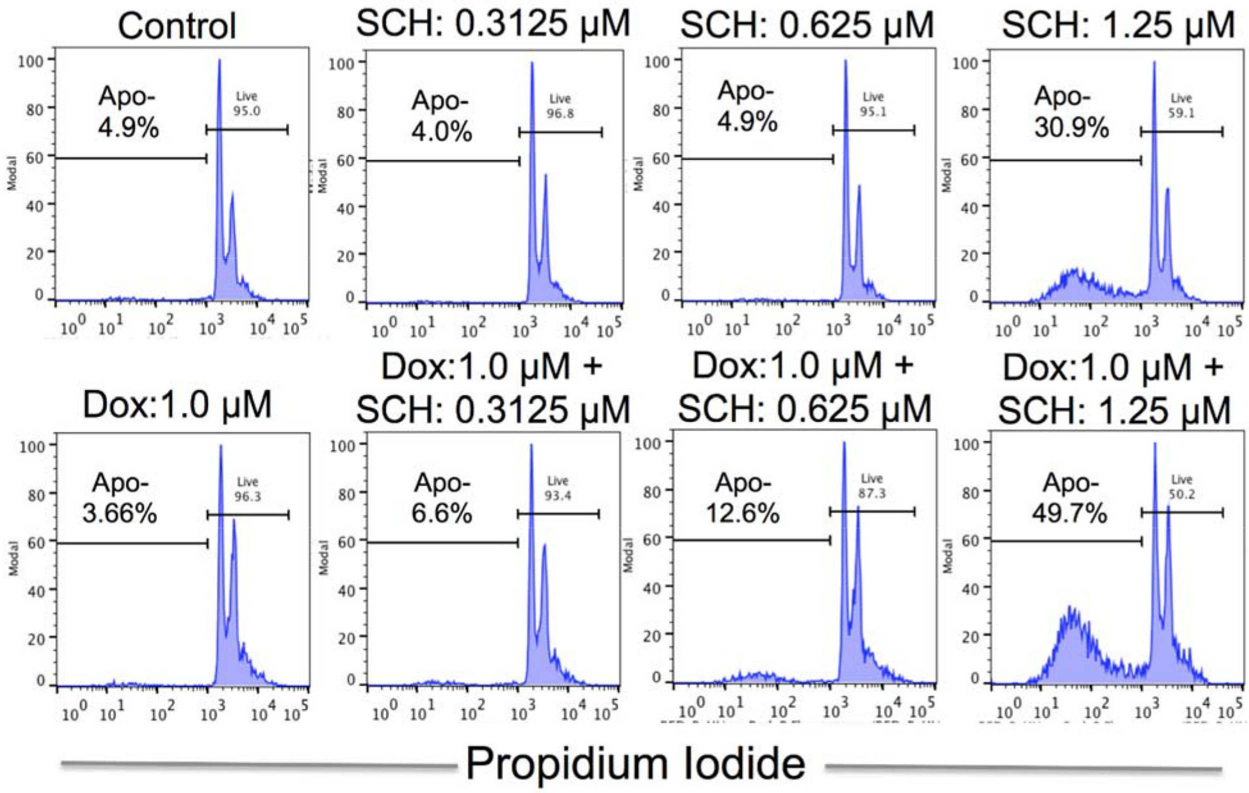

Supplementary Figure 7: FACS analysis results of U87MG cell endogenously wild-type for p53 expression evaluated for its response to Doxorubicin (1 μM) in the presence of different concentrations of SCH529074 (0.3125 to 1.25 μM), for measuring response to combination treatment.

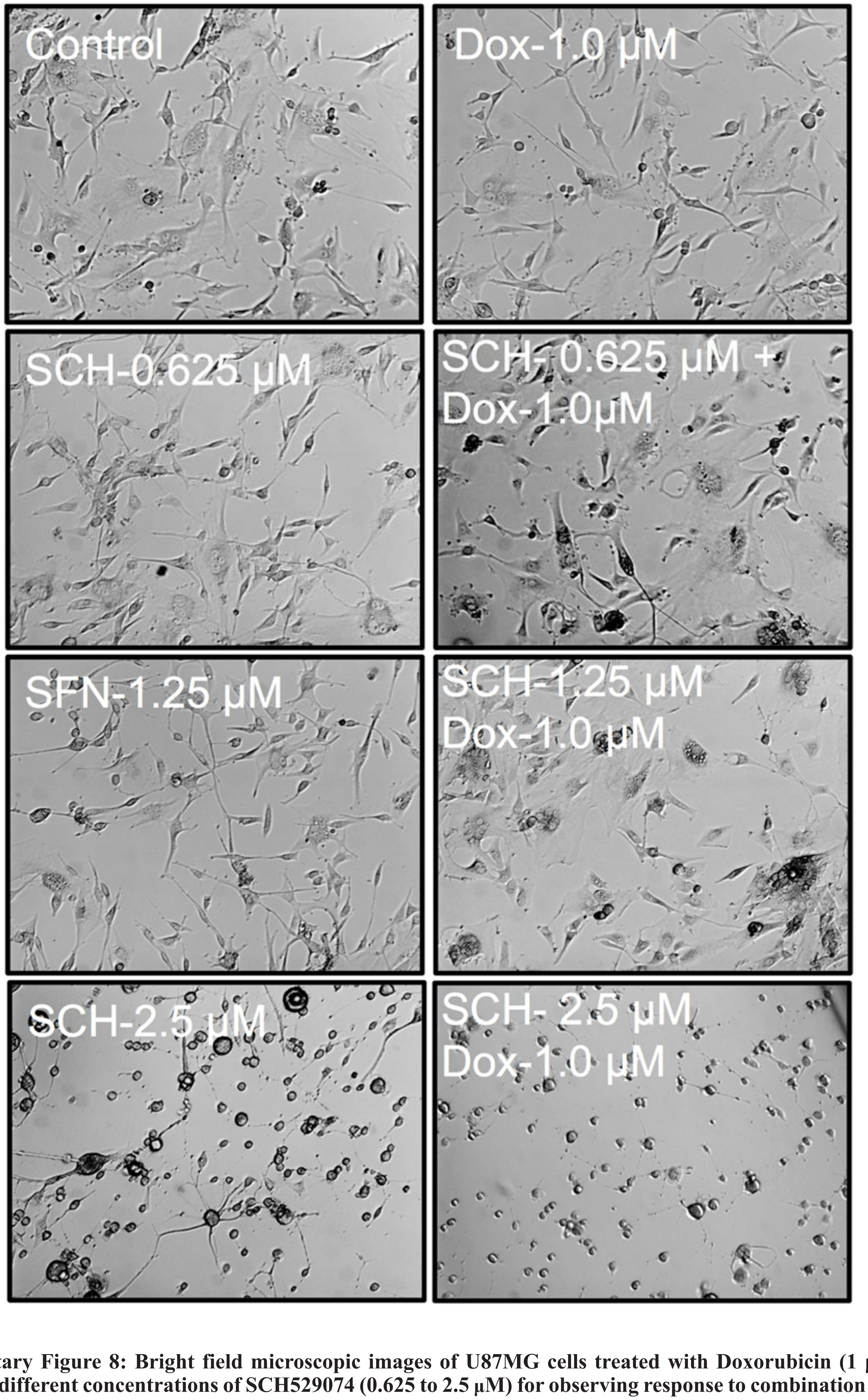

Supplementary Figure 8: Bright field microscopic images of U87MG cells treated with Doxorubicin (1 µM) in the presence of different concentrations of SCH529074 (0.625 to 2.5 µM) for observing response to combination treatment.

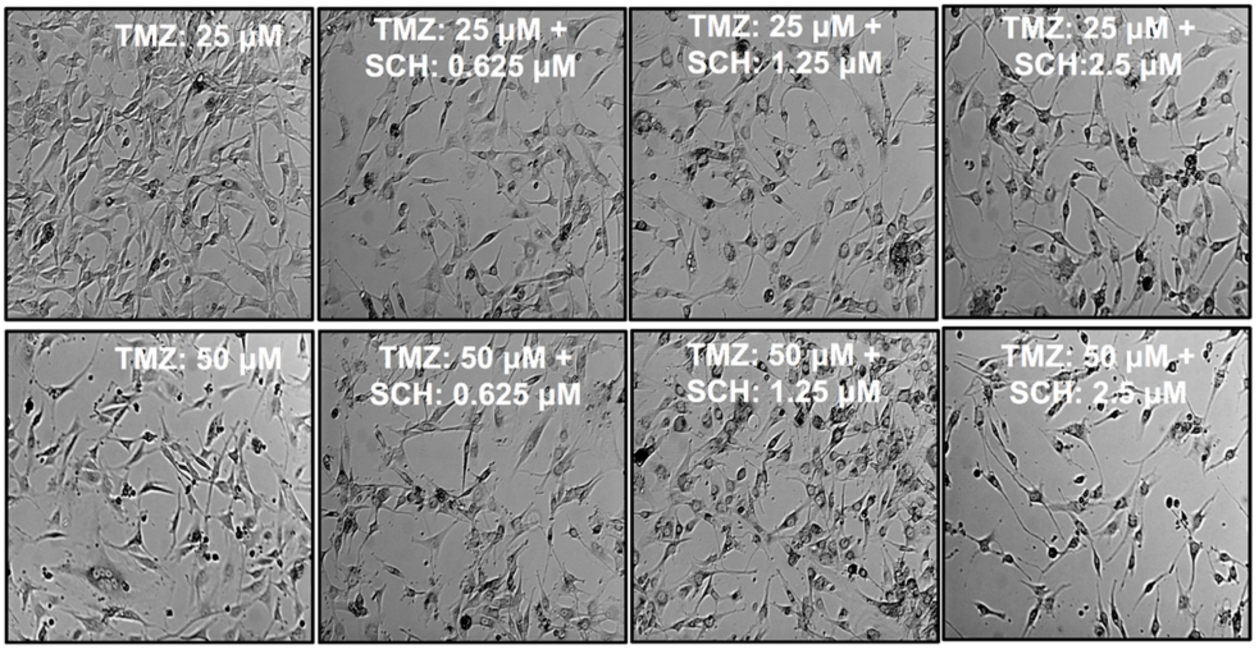

Supplementary Figure 9: Bright field microscopic images of U87MG cells treated with different concentrations of TMZ (25 and 50 µM) in the presence of different concentrations of SCH529074 (0.3125 to 1.25 µM), for observing response to combination treatment.
